# Intake of meat, meat mutagens, and iron and the risk of breast cancer in the Prostate, Lung, Colorectal, and Ovarian Cancer Screening Trial

**DOI:** 10.1038/sj.bjc.6605118

**Published:** 2009-06-09

**Authors:** L M Ferrucci, A J Cross, B I Graubard, L A Brinton, C A McCarty, R G Ziegler, X Ma, S T Mayne, R Sinha

**Affiliations:** 1 Division of Cancer Epidemiology and Genetics, National Cancer Institute, National Institutes of Health, Department of Health and Human Services, 6120 Executive Boulevard, Rockville, MD 20852, USA; 2 Yale School of Public Health, 60 College Street, New Haven, CT 06520, USA; 3 Marshfield Clinic Research Foundation, 1000 North Oak Avenue ML-1, Marshfield, WI 54449, USA

**Keywords:** breast cancer, meat, iron, epidemiology

## Abstract

**Background:**

Epidemiological evidence on meat intake and breast cancer is inconsistent, with little research on potentially carcinogenic meat-related exposures. We investigated meat subtypes, cooking practices, meat mutagens, iron, and subsequent breast cancer risk.

**Methods:**

Among 52 158 women (aged 55–74 years) in the Prostate, Lung, Colorectal, and Ovarian Cancer Screening Trial, who completed a food frequency questionnaire, 1205 invasive breast cancer cases were identified. We estimated meat mutagen and haem iron intake with databases accounting for cooking practices. Using Cox proportional hazards regression, we calculated hazard ratios (HRs) and 95% confidence intervals (CIs) within quintiles of intake.

**Results:**

Comparing the fifth to the first quintile, red meat (HR=1.23; 95% CI=1.00–1.51, *P* trend=0.22), the heterocyclic amine (HCA), 2-amino-3,8-dimethylimidazo[4,5-*f*]quinoxaline (MeIQx), (HR=1.26; 95% CI=1.03–1.55; *P* trend=0.12), and dietary iron (HR=1.25; 95% CI=1.02–1.52; *P* trend=0.03) were positively associated with breast cancer. We observed elevated, though not statistically significant, risks with processed meat, the HCA 2-amino-3,4,8-trimethylimidazo[4,5-*f*]quinoxaline (DiMeIQx), mutagenic activity, iron from meat, and haem iron from meat.

**Conclusion:**

In this prospective study, red meat, MeIQx, and dietary iron elevated the risk of invasive breast cancer, but there was no linear trend in the association except for dietary iron.

Evidence of the role of meat intake in breast cancer is mixed. There are conflicting findings from a pooled analysis of cohort studies (Missmer *et al*, 2002), and a meta-analysis of cohort and case–control studies (Boyd *et al*, 2003), yet several recent studies indicate that meat may increase the risk of postmenopausal breast cancer (Shannon *et al*, 2003; Steck *et al*, 2007; Taylor *et al*, 2007; Egeberg *et al*, 2008; Hu *et al*, 2008); meat-related exposures potentially involved, however, have not been well studied.

Meat mutagens, such as heterocyclic amines (HCAs) and polycyclic aromatic hydrocarbons (PAHs), are formed in meat cooked to well done at high temperatures (Sinha *et al*, 2005). Haem iron, found mainly in red meat, is more bioavailable than non-haem iron and, although iron homoeostasis is tightly controlled, haem iron absorption is less well regulated (Carpenter and Mahoney, 1992). Meat mutagens induce (el-Bayoumy *et al*, 1995; Snyderwine *et al*, 2002) and iron promotes (Thompson *et al*, 1991; Diwan *et al*, 1997) mammary carcinogenesis in rodent studies. Iron may also be involved in breast cancer through interaction with catechol oestrogen metabolites or production of hydroxyl radicals (Liehr and Jones, 2001; Huang, 2003; Kabat and Rohan, 2007). Limited epidemiological studies of meat mutagens (De Stefani *et al*, 1997; Delfino *et al*, 2000; Sinha *et al*, 2000; Steck *et al*, 2007; Sonestedt *et al*, 2008; Kabat *et al*, 2009; Mignone *et al*, 2009) and haem iron (Lee *et al*, 2004; Kabat *et al*, 2007) are inconsistent.

We used unique databases and a detailed meat questionnaire to comprehensively assess meat intake and potentially carcinogenic meat-related exposures in relation to postmenopausal invasive breast cancer in the prospective Prostate, Lung, Colorectal, and Ovarian (PLCO) Cancer Screening Trial.

## Materials and methods

The PLCO Cancer Screening Trial is a multi-center, randomised controlled trial designed to evaluate screening methods for the early detection of prostate, lung, colorectal, and ovarian cancer (Prorok *et al*, 2000). Briefly, 154 952 participants (78 217 women), aged 55–74 years, were recruited from 10 centres in the US between 1993 and 2001. On enrolment, the participants in the screened and non-screened arms of the trial completed a self-administered baseline questionnaire on demographics, personal/family cancer history, medical history, and lifestyle habits. Starting in 1998, diet was assessed using the Diet History Questionnaire (DHQ) (http://riskfactor.cancer.gov/DHQ/), the National Cancer Institute's (NCI) self-administered validated food frequency questionnaire (FFQ) (Subar *et al*, 2001). Each year, participants were sent annual study update questionnaires that asked whether they had been diagnosed with cancer by a health care provider. The study was approved by the institutional review boards at the NCI and PLCO study centres. All participants provided written informed consent.

Women were excluded from this analysis if they lacked the baseline questionnaire (*n*=2095) or the DHQ (*n*=16 886); missed more than seven food items on the DHG (*n*=1563); reported energy intake in the top or bottom 1% of women (*n*=1226); had a history of cancer other than non-melanoma skin cancer before dietary assessment (*n*=6819); or no follow-up time (*n*=1110). After exclusions, with some subjects meeting multiple criteria, our analytic cohort consisted of 52 158 women. The vast majority of the cohort was postmenopausal based on self-reported age at last period and reason for last period. Menopausal status was ambiguous for 1.7%, but 89.1% of the women with ambiguous data were 57 or older, so the cohort was assumed to be postmenopausal.

Incident invasive breast cancer cases were identified through self-report from the annual study update questionnaire, physician reports, or through reports from the next of kin. This analysis includes only histologically confirmed invasive breast cancers based on pathology reports and medical records. Oestrogen receptor (ER) and progesterone receptor (PR) data collection is ongoing; we had receptor status for 388 of our cases. Entry date for the analytic cohort was the latest of the following: randomisation, completion of baseline questionnaire, or completion of the DHQ. Follow-up ended on 31 December 2006, with breast cancer cases exiting at the date of diagnosis and non-cases exiting at the date of the most recent annual study update questionnaire without a report of breast cancer.

The DHQ assessed usual intake (frequency and portion size) of 124 food items over the past year. Nutrient intake was estimated using the Diet^*^Calc Analysis Program (version 1.4.3, National Cancer Institute, Applied Research Program, 2005). Red meat (g per day) included bacon, beef, cheeseburgers, cold cuts, ham, hamburgers, hot dogs, liver, pork, sausage, veal, venison, and red meat from mixed dishes. White meat included chicken, fish, and turkey. Processed meat included bacon, cold cuts, ham, hot dogs, and sausage.

With the Computerised Heterocyclic Amines Resource for Research in Epidemiology of Disease (CHARRED) (http://www.charred.cancer.gov) software application and data from a detailed meat-cooking module included in the DHQ, we generated intake estimates of three HCAs (ng per day): 2-amino-3,4,8-trimethylimidazo[4,5-*f*]quinoxaline (DiMeIQx), 2-amino-3,8-dimethylimidazo[4,5-*f*]quinoxaline (MeIQx), and 2-amino-1-methyl-6-phenyl-imidazo[4,5-*b*]pyridine (PhIP), as well as benzo[*a*]pyrene (B[*a*]P), a marker of total PAH exposure, and mutagenic activity in meat (revertant colonies per day) (Sinha *et al*, 2005). We estimated haem iron from meat using the NCI heme iron database based on the measured values of haem iron from meat samples cooked by a range of methods to varying doneness levels (Sinha *et al*, 2005). The United States Department of Agriculture (USDA, 2007) Survey Nutrient Database was used to estimate iron from meat (limited to meats in the haem iron database).

### Statistical analysis

Hazard ratios (HRs) and 95% confidence intervals (CIs) were estimated using Cox proportional hazards regression with age at baseline as the underlying time metric; proportional hazards assumptions were not violated. Quintile cut points for the dietary exposures were based on intake in the analytic cohort, with the lowest quintile as referent. Dietary variables, except the meat mutagens, were energy adjusted using the multivariate nutrient density method; residual adjustment did not alter our findings (Willett, 1998). Tests for linear trend were based on median values of each quintile. *P*-values are two-sided and analyses were conducted using SAS (SAS Institute, Cary, NC, USA).

Multivariate models were adjusted for the following potential confounders, which were selected because inclusion in the age-adjusted model resulted in a 10% change in risk estimates, they were associated with breast cancer in this dataset, or are established breast cancer risk factors: age, race, education, study centre, randomisation group, family history of breast cancer, age at menarche, age at menopause (natural or surgical reasons), age at first birth and number of live births, history of benign breast disease, number of mammograms during past 3 years, menopausal hormone therapy, body mass index (BMI), and intakes of alcohol, total fat, and total energy. We included cross product terms in the multivariate models to assess effect modification by alcohol intake, parity, family history of breast cancer, BMI, menopausal hormone therapy, and number of mammograms.

## Results

During a mean follow-up of 5.5 years, we identified 1205 invasive breast cancer cases. Mean total meat intake was 62.9 g per 1000 kcal, with 29.5 g per 1000 kcal from red meat and 33.4 g per 1 000 kcal from white meat. Women in the highest quintile of red meat intake were slightly younger, less educated, and were less likely to have a family history of breast cancer or more than one mammogram in the past 3 years than those in the lowest quintile (Table 1). Furthermore, women consuming the most red meat were more likely to have a higher BMI, to have used oral contraceptives, to be current smokers, and to have higher energy and fat intakes. Similar patterns were seen for age, BMI, oral contraceptives, and total energy intake comparing women in the highest quintile of white meat intake to those in the lowest quintile. In contrast, high white meat consumers were more educated and less likely to be current smokers than those consuming the least white meat.

We observed statistically significant or borderline-positive associations between red meat and breast cancer starting in the second quintile, and there was no evidence for a dose–response effect (*P* trend=0.22), consistent with a potential threshold effect (Table 2). When we compared ER-positive/PR-positive tumours (*n*=259) to non-cases, the effect of red meat seemed to be stronger (Q5 *vs* Q1 H=1.59; 95% CI=1.03–2.48; *P* trend=0.09). There were no statistically significant associations with processed meat, white meat, or individual meat items.

Pan-fried meat, grilled meat, well/very well done meat, PhIP and B[*a*]P were not associated with breast cancer (Table 3). There was a borderline statistically significant increased risk for women consuming the most meats that were cooked well or very well done by pan-frying or grilling compared with those consuming the least. Women in the highest quintile of MeIQx compared with those in lowest had statistically significant elevated risks of breast cancer. We also observed a marginally significant increased risk of breast cancer for women with the highest intakes of DiMeIQx and mutagenic activity; both associations had a statistically significant linear trend.

Dietary iron was positively associated with breast cancer in a dose–response manner, yet there was no association for total iron or iron from supplements (Table 4). There were suggestive positive associations with iron from meat and haem iron from meat, but these relationships failed to reach statistical significance in the top quintiles.

We found some evidence of a stronger effect of red meat (*P* interaction=0.083), PhIP (0.014), mutagenic activity (0.010), and haem iron (0.016) on breast cancer risk in women with BMI <25 (469 cases) as opposed to those who were overweight (BMI 25–<30, 444 cases) or obese (BMI ⩾30, 292 cases) (data not shown). There was no effect modification by alcohol intake, parity, family history of breast cancer, menopausal hormone therapy, or number of mammograms.

## Discussion

In this prospective study, we observed positive associations between red meat, MeIQx and dietary iron, and invasive post-menopausal breast cancer. Only the dietary iron association was statistically significant for linear trend, as the associations with red meat and MeIQx were more consistent with a threshold effect starting in the second quintile. We also found elevated, though not statistically significant, risk of breast cancer with intakes of DiMeIQx, mutagenic activity, iron from meat, and haem iron from meat.

Our results support an association between red meat and post-menopausal breast cancer, and are similar to two other recent prospective studies (Taylor *et al*, 2007; Egeberg *et al*, 2008). Our finding of a potentially stronger effect of red meat among women with ER-positive/PR-positive tumours is similar to results in pre-menopausal women for adolescent (Linos *et al*, 2008) and recent (Cho *et al*, 2006) red meat intake. However, epidemiological evidence on red meat and breast cancer is mixed, with null results in four other recent prospective studies (Holmes *et al*, 2003; van der Hel *et al*, 2004; Kabat *et al*, 2007, 2009). It is not clear why prospective investigations of meat and breast cancer have been inconsistent, as the potential mechanisms underlying the association should not vary in different populations. The variation could be in part due to the complexity of assessing meat intake. Our observation of a potential threshold effect is intriguing and may indicate that those consuming the least red meat are different from other women.

Similar to some (Gertig *et al*, 1999; Delfino *et al*, 2000; Kabat *et al*, 2009), but not all previous studies (De Stefani *et al*, 1997; Zheng *et al*, 1998; Dai *et al*, 2002; Steck *et al*, 2007), we did not observe a clear association with well/very well done and high temperature cooked meats. Our finding for MeIQx is supported by one previous case–control study (De Stefani *et al*, 1997); nevertheless, other studies of meat mutagens have been null (Delfino *et al*, 2000; Steck *et al*, 2007; Sonestedt *et al*, 2008; Kabat *et al*, 2009; Mignone *et al*, 2009). Interactions between meat (Zheng *et al*, 1999; Egeberg *et al*, 2008) or well-done meat (Zheng *et al*, 1999; Deitz *et al*, 2000) and xenobiotic-metabolising gene variants might explain these contradictory findings.

Epidemiological studies of iron and breast cancer are also mixed. Three case–control studies of dietary iron (Levi *et al*, 2001; Adzersen *et al*, 2003; Kallianpur *et al*, 2008) have been null, yet two others have found inverse associations (Negri *et al*, 1996; Cade *et al*, 1998). One cohort study reported positive associations for both dietary iron and haem iron among high alcohol consumers (Lee *et al*, 2004), yet another cohort found no association with either exposure regardless of alcohol intake (Kabat *et al*, 2007). Future research may need to evaluate the role of genes involved in iron absorption or oxidative stress, as breast cancer risk may differ by certain gene variants (Kallianpur *et al*, 2004; Hong *et al*, 2007).

Our results for haem iron may vary from earlier research (Lee *et al*, 2004; Kabat *et al*, 2007) that calculated haem iron as standard proportions of total iron from meat (Monsen and Balintfy, 1982; Balder *et al*, 2006), as we estimated haem iron based on measured values from meat samples. The pattern of risk that we observed for haem iron was similar to that for red meat. As these measures are highly correlated (*r*=0.77) they cannot be evaluated in the same model, making it difficult to determine whether the risk can be attributed to this individual component. However, individuals were not always ranked in the same quintile of the two exposures (weighted Kappa=0.59), indicating that haem iron may not just be a surrogate for red meat.

Strengths of this analysis include the detailed prospective data and investigation of specific meat-related exposures with separate, plausible pathways for influencing breast carcinogenesis. Although there is inherent measurement error in FFQs, this typically results in attenuation of risks. To minimise this error, our models included total energy intake. However, our ability to detect small associations may still be limited. The diet was assessed only once and this may not have been the time period most relevant to neoplasia. We also had limited ability to evaluate tumour sub-types, as our results were based on ongoing and therefore incomplete ascertainment. Finally, the observed associations could be due to unmeasured confounding, although we investigated many potential confounders.

Overall, the epidemiological evidence for meat in relation to breast cancer remains inconclusive; however, with few known modifiable risk factors, this dietary component should be further investigated. Our results regarding meat mutagens and haem iron indicate a need to evaluate multiple meat-related exposures in relation to breast carcinogenesis.

## Figures and Tables

**Table 1:** Means and proportions^a^ of baseline characteristics by red meat and white meat quintiles (*n*=52 158)

	**Quintile red meat**			**Quintile white meat**		
**Characteristic**	**1**	**3**	**5**	**1**	**3**	**5**
Quintile mean (g per 1000 kcal)	8.8	26.7	57.1	8.7	26.2	75.5
Age (years)	66.0	65.3	64.5	66.1	65.3	64.4
						
*Race* (%)						
Non-Hispanic white	85.1	92.6	93.9	91.0	92.3	88.7
Non-Hispanic black	7.0	3.1	2.2	3.7	3.1	5.6
Hispanic	1.3	1.2	1.3	1.5	1.2	1.2
Asian	5.9	2.6	1.9	3.2	2.8	3.7
						
College graduate or postgraduate (%)	38.8	31.4	22.9	24.2	31.4	36.7
Family history of breast cancer (%)	14.1	14.1	13.6	14.1	13.8	13.7
History of benign breast disease (%)	28.7	28.6	26.7	26.0	28.4	30.4
Body mass index (kg m^−2^)	25.7	27.1	28.4	26.8	27.1	27.4
Height (inches)	64.2	64.3	64.3	64.2	64.3	64.3
						
*Age at menarche (years)* (%)						
<10	1.5	1.3	1.8	1.3	1.5	1.7
10–11	18.2	18.0	19.4	17.0	18.8	20.8
12–13	54.5	54.6	53.8	53.3	53.6	54.6
14–15	20.8	21.8	20.3	22.8	21.7	19.0
⩾16	4.8	4.2	4.5	5.5	4.2	3.9
Oral contraceptive use, ever (%)	51.9	55.7	58.8	51.6	55.6	60.0
						
*Age at first birth (years)* (%)						
<19	13.4	16.0	18.9	18.5	16.0	14.8
20–24	44.0	47.9	49.9	46.7	48.1	47.1
25–29	23.1	20.0	17.4	18.6	20.4	21.4
⩾30	8.1	7.1	5.8	6.8	6.4	7.0
						
*Parity* (%)						
Nulliparous	11.1	8.6	7.7	9.1	8.8	9.3
1	7.9	6.7	6.7	7.3	6.3	8.0
2	25.5	23.6	22.9	22.0	23.9	26.2
3	24.4	24.6	25.9	23.6	25.6	25.6
⩾4	31.0	36.3	36.7	37.9	35.3	30.9
>1 mammogram in past 3 years (%)	74.8	73.6	69.5	68.2	73.5	76.7
						
*Age at menopause (years)* (%)						
<40	13.3	13.2	14.4	14.7	13.4	13.6
40–44	13.2	14.2	14.7	14.5	13.7	12.9
45–49	23.7	23.5	22.8	23.6	23.4	23.2
50–54	38.1	36.2	36.5	35.9	37.0	38.0
⩾55	11.1	12.2	11.0	10.6	11.8	11.5
						
*Menopausal hormone therapy* (%)						
Never	31.7	31.1	33.7	34.9	31.7	29.1
<1 year	11.6	11.2	10.6	11.3	11.1	11.7
1–5 years	19.4	19.3	18.6	17.6	18.8	20.7
6–9 years	12.1	13.0	12.9	10.9	13.3	14.3
⩾10 years	25.1	25.4	24.2	25.3	25.2	24.3
						
*Smoking* (%)						
Never	59.2	58.5	54.3	59.4	57.8	53.6
Former	35.4	33.7	33.8	30.1	34.4	39.0
Current	5.4	7.8	11.9	10.5	7.8	7.4
						
Total caloric intake (kcal per day)	1476	1495	1547	1491	1481	1528
Total meat (g per 1000 kcal)	44.0	59.2	90.8	34.2	58.0	103.7
Total fat (g per 1000 kcal)	30.0	34.9	39.5	34.4	34.9	34.8
Alcohol (g per day)	4.7	5.4	4.7	4.6	5.2	5.0

a May not sum to 100% due to missing data or rounding.

**Table 2:** Distribution and HRs^a^ with 95% CIs for breast cancer risk according to quintiles of meat (g per 1000 kcal)

**Characteristic**	**Q1**	**Q2**	**Q3**	**Q4**	**Q5**	***P* trend^b^**
*Red meat*						
Cases/Person-years	215/58 396	280/57 813	228/57 651	239/57 280	243/57 108	
Median (range)	9.4 (⩽14.6)	18.8 (>14.6–22.8)	26.7 (>22.8–31.0)	36.1 (>31.0–42.5)	52.8 (>42.5–196.3)	
HR (95% CI)	1.00	1.32 (1.10–1.58)	1.09 (0.90–1.32)	1.16 (0.96–1.42)	1.23 (1.00–1.51)	0.22
Steak						
Cases/Person-years	234/57 950	239/58 452	229/57 570	261/57 356	242/56 921	
Median (range)	0.0 (⩽0.6)	0.9 (>0.6–1.2)	1.7 (>1.2–2.4)	3.2 (>2.4–4.7)	7.5 (>4.7–131.3)	
HR (95% CI)	1.00	0.94 (0.78–1.14)	0.89 (0.73–1.08)	1.01 (0.83–1.23)	0.95 (0.78–1.17)	0.82
Hamburger						
Cases/Person-years	209/58 291	269/57 747	244/57 809	255/57 186	228/57 215	
Median (range)	0.3 (⩽0.6)	1.1 (>0.6–1.6)	2.0 (>1.6–2.7)	3.6 (>2.7–4.9)	7.4 (>4.9–98.8)	
HR (95% CI)	1.00	1.28 (1.06–1.55)	1.13 (0.92–1.38)	1.22 (1.00–1.50)	1.10 (0.89–1.37)	0.84
Sausage						
Cases/Person-years	251/63 770	221/52 321	232/58 096	241/57 370	260/56 690	
Median (range)	0.0 (⩽0.0)	0.1 (>0.0–0.2)	0.3 (>0.2–0.4)	0.7 (>0.4–1.0)	1.7 (>1.0–97.5)	
HR (95% CI)	1.00	1.05 (0.87–1.26)	0.98 (0.82–1.19)	1.04 (0.86–1.26)	1.14 (0.94–1.39)	0.10
Bacon						
Cases/Person-years	233/58 521	253/58 198	229/57 649	237/57 913	253/56 687	
Median (range)	0.0 (⩽0.1)	0.1 (>0.1–0.2)	0.3 (>0.2–0.4)	0.6 (>0.4–1.0)	1.7 (>1.0–46.2)	
HR (95% CI)	1.00	1.06 (0.88–1.28)	0.95 (0.78–1.16)	0.99 (0.81–1.21)	1.07 (0.87–1.32)	0.38
Pork chops						
Cases/Person-years	223/58 109	242/57 656	245/57 695	255/57 625	240/57 163	
Median (range)	0.1 (⩽0.4)	0.6 (>0.4–0.9)	1.2 (>0.9–1.6)	2.1 (>1.6–3.0)	4.5 (>3.0–74.2)	
HR (95% CI)	1.00	1.03 (0.86–1.25)	1.01 (0.83–1.23)	1.06 (0.86–1.29)	0.99 (0.80–1.22)	0.94
						
*White meat*						
Cases/Person-years	223/57 645	246/57 909	261/57 593	236/57 727	239/57 375	
Median (range)	9.2 (⩽13.4)	17.4 (>13.4–21.5)	26.1 (>21.5–31.4)	38.6 (>31.4–49.1)	66.9 (>49.1–313.6)	
HR (95% CI)	1.00	1.06 (0.88–1.27)	1.11 (0.93–1.33)	0.99 (0.82–1.19)	1.01 (0.84–1.22)	0.70
Chicken						
Cases/Person-years	235/57 630	243/57 859	244/57 877	249/57 445	234/57 437	
Median (range)	3.6 (⩽6.0)	8.4 (>6.0–11.2)	14.4 (>11.2–18.3)	23.7 (>18.3–32.5)	48.8 (>32.5–261.8)	
HR (95% CI)	1.00	0.98 (0.81–1.18)	0.96 (0.80–1.16)	0.98 (0.81–1.18)	0.93 (0.77–1.13)	0.52
Fish						
Cases/Person-years	218/57 510	248/57 769	249/57 551	241/57 925	249/57 494	
Median (range)	1.8 (⩽3.1)	4.2 (>3.1–5.4)	6.8 (>5.4–8.5)	10.6 (>8.5–14.0)	20.4 (>14.0–229.4)	
HR (95% CI)	1.00	1.10 (0.92–1.33)	1.09 (0.90–1.31)	1.04 (0.86–1.26)	1.08 (0.89–1.31)	0.76
						
*Processed meat*						
Cases/Person-years	218/58 209	250/57 934	251/57 597	255/57 278	231/57 230	
Median (range)	1.4 (⩽2.4)	3.4 (>2.4–4.3)	5.5 (>4.3–6.9)	8.7 (>6.9–11.6)	16.9 (>11.6–124.1)	
HR (95% CI)	1.00	1.17 (0.98–1.41)	1.20 (0.99–1.45)	1.23 (1.02–1.49)	1.12 (0.92–1.36)	0.66

Abbreviations: CI=confidence interval; HR=hazard ratio; Q=quintile.

a Adjusted for age (continuous), race (non-Hispanic white, non-Hispanic black, Hispanic, Asian, other), education (⩽11 years, 12 years or high school graduate, post high school training, some college, college graduate or postgraduate education, missing), study centre, randomisation group, family history of breast cancer (yes, no, missing), age at menarche (<10, 10–11, 12–13, 14–15, ⩾16 years, missing), age at menopause (<40, 40–44, 45–49, 50–54, ⩾55 years, missing), age at first birth and number of live births (nulliparous; <20 years and 1, 2, or ⩾3 births; 20–29 years and 1, 2, or ⩾3 births; ⩾30 years and 1, 2, or ⩾3 births; missing), history of benign breast disease (yes, no, missing), number of mammograms during past 3 years (0, 1, >1, missing), menopausal hormone therapy use (never, ⩽1, 2–5, 6–9, ⩾10 years), body mass index (18.5–24.9, 25–29.9, ⩾30 kg m^−2^), alcohol intake (<5, 5–14.9, ⩾15 g per day), total fat intake (continuous, g per 1000 kcal), and total energy intake (continuous, kcal per day).

b *P* trend calculated using the median of each quintile.

**Table 3:** Distribution and HRs^a^ with 95% CIs for breast cancer risk according to quintiles of cooking method, doneness level, and meat mutagens

**Characteristic**	**Q1**	**Q2**	**Q3**	**Q4**	**Q5**	**P trend^b^**
*Cooking method and doneness*						
Pan-fried meat						
Cases/Person-years	233/56 615	243/57 881	248/57 319	245/57 316	235/57 245	
Median (range) (g per 1000 kcal)	0.1 (⩽0.4)	0.7 (>0.4–1.1)	1.7 (>1.1–2.4)	3.4 (>2.4–5.0)	8.1 (>5.0–77.1)	
HR (95% CI)	1.00	1.01 (0.84–1.21)	1.05 (0.87–1.27)	1.04 (0.85–1.27)	1.03 (0.84–1.27)	0.76
Grilled meat						
Cases/Person-years	222/56 653	246/58 049	252/57 887	236/57 289	248/56 498	
Median (range) (g per 1000 kcal)	0.1 (⩽0.7)	1.2 (>0.7–1.9)	2.8 (>1.9–3.9)	5.4 (>3.9–7.6)	11.9 (>7.6–161.1)	
HR (95% CI)	1.00	1.07 (0.89–1.29)	1.09 (0.90–1.31)	1.02 (0.84–1.24)	1.10 (0.90–1.34)	0.54
Well/very well done meat						
Cases/Person-years	235/56 101	235/57 952	239/57 865	236/57 662	259/56 796	
Median (range) (g per 1000 kcal)	1.9 (⩽3.2)	4.4 (>3.2–5.7)	7.1 (>5.7–8.9)	11.1 (>8.9–14.3)	20.2 (>14.3–158.8)	
HR (95% CI)	1.00	0.94 (0.78–1.13)	0.96 (0.79–1.15)	0.95 (0.78–1.15)	1.09 (0.90–1.32)	0.17
Grilled/pan fried well/very well done meat						
Cases/Person-years	227/56 511	250/58 079	249/57 823	215/57 470	263/57 493	
Median (range) (g per 1000 kcal)	0.3 (⩽0.9)	1.5 (>0.9–2.3)	3.1 (>2.3–4.2)	5.7 (>4.2–7.7)	11.6 (>7.7–158.8)	
HR (95% CI)	1.00	1.07 (0.89–1.29)	1.07 (0.89–1.29)	0.94 (0.77–1.14)	1.20 (0.99–1.45)	0.10
						
*Meat mutagens*						
DiMeIQx						
Cases/Person-years	236/58 051	236/57 899	227/57 868	245/57 303	261/57 127	
Median (range) (ng per day)	0.0 (⩽0.1)	0.2 (>0.1–0.3)	0.5 (>0.3–0.7)	1.0 (>0.7–1.6)	2.5 (>1.6–76.2)	
HR (95% CI)	1.00	1.03 (0.86–1.24)	0.99 (0.83–1.20)	1.08 (0.90–1.30)	1.18 (0.98–1.42)	0.04
MeIQx						
Cases/Person-years	220/58 585	255/58 100	245/57 333	241/57 223	244/57 007	
Median (range) (ng per day)	1.5 (⩽2.8)	4.2 (>2.8–5.7)	7.6 (>5.7–10.0)	13.3 (>10.0–18.2)	27.8 (>18.2–516.2)	
HR (95% CI)	1.00	1.20 (1.00–1.43)	1.18 (0.98–1.43)	1.17 (0.97–1.42)	1.26 (1.03–1.55)	0.12
PhIP						
Cases/Person-years	238/58 305	224/57 940	255/57 611	235/57 528	253/56 865	
Median (range) (ng per day)	3.7 (⩽7.6)	12.5 (>7.6–18.7)	25.9 (>18.7–36.8)	51.0 (>36.8–75.2)	121.1 (>75.2–3178.8)	
HR (95% CI)	1.00	0.95 (0.79–1.15)	1.09 (0.91–1.31)	1.00 (0.83–1.21)	1.11 (0.92–1.34)	0.22
B[*a*]P						
Cases/Person-years	234/58 269	257/58 144	244/57 638	244/57 278	226/56 918	
Median (range) (ng per day)	0.5 (⩽1.3)	2.2 (>1.3–3.7)	5.4 (>3.7–7.4)	11.9 (>7.4–16.7)	29.9 (>16.7–697.7)	
HR (95% CI)	1.00	1.11 (0.93–1.33)	1.08 (0.90–1.30)	1.08 (0.90–1.30)	1.01 (0.83–1.23)	0.59
Mutagenic activity						
Cases/Person-years	231/58 649	241/57 870	212/57 517	274/57 358	247/56 854	
Median (range) (revertant colonies per day)	235 (⩽448)	672 (>448–925)	1247 (>925–1640)	2205 (>1640–3031)	4691 (>3031–225039)	
HR (95% CI)	1.00	1.08 (0.90–1.30)	0.96 (0.80–1.17)	1.26 (1.05–1.52)	1.18 (0.97–1.44)	0.05

Abbreviations: B[*a*]P=benzo[*a*]pyrene; CI=confidence interval; DiMeIQx=2-amino-3,4,8-trimethylimidazo[4,5-*f*]quinoxaline; HR=hazard ratio; MeIQx=2-amino-3,8-dimethylimidazo[4,5-*f*]quinoxaline; PhIP=2-amino-1-methyl-6-phenyl-imidazo[4,5-*b*]pyridine; Q=quintile.

a Adjusted for age (continuous), race (non-Hispanic white, non-Hispanic black, Hispanic, Asian, other), education (⩽11 years, 12 years or high school graduate, post high school training, some college, college graduate or postgraduate education, missing), study centre, randomisation group, family history of breast cancer (yes, no, missing), age at menarche (<10, 10–11, 12–13, 14–15, ⩾16 years, missing), age at menopause (<40, 40–44, 45–49, 50–54, ⩾55 years, missing), age at first birth and number of live births (nulliparous; <20 years and 1, 2, or ⩾3 births; 20–29 years and 1, 2, or ⩾3 births; ⩾30 years and 1, 2, or ⩾3 births; missing), history of benign breast disease (yes, no, missing), number of mammograms during past 3 years (0, 1, >1, missing), menopausal hormone therapy use (never, ⩽1, 2–5, 6–9, ⩾10 years), body mass index (18.5–24.9, 25–29.9, ⩾30 kg m^−2^), alcohol intake (<5, 5–14.9, ⩾15 g per day), total fat intake (continuous, g per 1000 kcal), and total energy intake (continuous, kcal per day).

b *P* trend calculated using the median of each quintile.

**Table 4:** Distribution and HRs^a^ with 95% CIs for breast cancer risk according to iron and iron and haem iron from meat

**Characteristic**	**Q1**	**Q2**	**Q3**	**Q4**	**Q5**	***P* trend^b^**
*Total iron* c						
Cases/Person-years	216/57 408	247/58 320	242/57 471	246/57 486	254/57 564	
Median (range) (mg per day)	9.6 (⩽11.4)	13.4 (>11.4–16.8)	25.5 (>16.8–28.0)	29.5 (>28.0–31.2)	33.7 (>31.2–68.6)	
HR (95% CI)	1.00	1.09 (0.90–1.31)	1.07 (0.89–1.29)	1.05 (0.87–1.27)	1.08 (0.90–1.30)	0.58
						
*Dietary iron*						
Cases/Person-years	216/56 980	240/57 697	228/57 697	247/57 853	274/58 021	
Median (range) (mg per 1000 kcal)	6.2 (⩽6.9)	7.4 (>6.9–7.8)	8.3 (>7.8–8.8)	9.5 (>8.8–10.3)	11.6 (>10.3–37.5)	
HR (95% CI)	1.00	1.09 (0.90–1.31)	1.03 (0.85–1.25)	1.12 (0.92–1.36)	1.25 (1.02–1.52)	0.03
						
*Iron from meat* d						
Cases/Person-years	218/58 422	256/58 105	258/57 650	237/57 174	236/56 898	
Median (range) (mg per 1000 kcal)	0.13 (⩽0.20)	0.26 (>0.20–0.31)	0.37 (>0.31–0.44)	0.51 (>0.44–0.61)	0.78 (>0.61–5.00)	
HR (95% CI)	1.00	1.19 (0.99–1.43)	1.21 (1.00–1.45)	1.14 (0.94–1.38)	1.16 (0.95–1.42)	0.37
						
*Haem iron from meat*						
Cases/Person-years	216/58 523	259/58 136	254/57 492	250/57 157	226/56 940	
Median (range) (mg per 1000 kcal)	0.05 (⩽0.07)	0.09 (>0.07–0.11)	0.14 (>0.11–0.16)	0.19 (>0.16–0.23)	0.29 (>0.23–1.49)	
HR (95% CI)	1.00	1.22 (1.02–1.47)	1.21 (1.01–1.46)	1.22 (1.01–1.47)	1.12 (0.92–1.38)	0.59
						
*Supplemental iron* e						
Cases/Person-years	463/113 215	696/163 714	46/11 320			
Range (mg per day)	0	0.3–18	21.4–39.4			
HR (95% CI)	1.00	0.99 (0.88–1.12)	1.00 (0.74–1.35)			0.94

Abbreviations: CI=confidence interval; HR=hazard ratio; Q=quintile.

a Adjusted for age (continuous), race (non-Hispanic white, non-Hispanic black, Hispanic, Asian, other), education (⩽11 years, 12 years or high school graduate, post high school training, some college, college graduate or postgraduate education, missing), study centre, randomisation group, family history of breast cancer (yes, no, missing), age at menarche (<10, 10–11, 12–13, 14–15, ⩾16 years, missing), age at menopause (<40, 40–44, 45–49, 50–54, ⩾55 years, missing), age at first birth and number of live births (nulliparous; <20 years and 1, 2, or ⩾3 births; 20–29 years and 1, 2, or ⩾3 births; ⩾30 years and 1, 2, or ⩾3 births; missing), history of benign breast disease (yes, no, missing), number of mammograms during past 3 years (0, 1, >1, missing), menopausal hormone therapy use (never, ⩽1, 2–5, 6–9, ⩾10 years), body mass index (18.5–24.9, 25–29.9, ⩾30 kg m^−2^), alcohol intake (<5, 5–14.9, ⩾15 g per day), total fat intake (continuous, g per 1000 kcal), and total energy intake (continuous, kcal per day).

b *P* trend calculated using the median of each quintile.

c Dietary iron (residual energy adjusted) plus iron from supplements. Nutrients in this model were residual energy adjusted.

d Limited to only those meats in the haem iron database.

e Owing to the small number of people taking supplements containing iron, iron from supplements was broken into three levels.
